# Cystatin F Depletion in *Mycobacterium tuberculosis*-Infected Macrophages Improves Cathepsin C/Granzyme B-Driven Cytotoxic Effects on HIV-Infected Cells during Coinfection

**DOI:** 10.3390/ijms25158141

**Published:** 2024-07-26

**Authors:** Manoj Mandal, David Pires, Marta Calado, José Miguel Azevedo-Pereira, Elsa Anes

**Affiliations:** 1 Host-Pathogen Interactions Unit, Research Institute for Medicines, iMed-ULisboa, Faculty of Pharmacy, Universidade de Lisboa, Av. Prof. Gama Pinto, 1649-003 Lisboa, Portugal; mmandal@ff.ulisboa.pt (M.M.); dpires@ff.ulisboa.pt (D.P.); miguel.pereira@ff.ulisboa.pt (J.M.A.-P.); 2 Center for Interdisciplinary Research in Health, Católica Medical School, Universidade Católica Portuguesa, Estrada Octávio Pato, 2635-631 Rio de Mouro, Portugal

**Keywords:** cystatin F, cytotoxic immune cells, cathepsin C, granzyme B, Mtb–HIV coinfection

## Abstract

Cystatin F (CstF) is a protease inhibitor of cysteine cathepsins, including those involved in activating the perforin/granzyme cytotoxic pathways. It is targeted at the endolysosomal pathway but can also be secreted to the extracellular milieu or endocytosed by bystander cells. CstF was shown to be significantly increased in tuberculous pleurisy, and during HIV coinfection, pleural fluids display high viral loads. In human macrophages, our previous results revealed a strong upregulation of CstF in phagocytes activated by interferon γ or after infection with *Mycobacterium tuberculosis* (Mtb). CstF manipulation using RNA silencing led to increased proteolytic activity of lysosomal cathepsins, improving Mtb intracellular killing. In the present work, we investigate the impact of CstF depletion in macrophages during the coinfection of Mtb-infected phagocytes with lymphocytes infected with HIV. The results indicate that decreasing the CstF released by phagocytes increases the major pro-granzyme convertase cathepsin C of cytotoxic immune cells from peripheral blood-derived lymphocytes. Consequently, an observed augmentation of the granzyme B cytolytic activity leads to a significant reduction in viral replication in HIV-infected CD4^+^ T-lymphocytes. Ultimately, this knowledge can be crucial for developing new therapeutic approaches to control both pathogens based on manipulating CstF.

## 1. Introduction

*Mycobacterium tuberculosis* (Mtb) and the human immunodeficiency virus (HIV) are syndemic interaction pathogens [1,2,3]. They synergize an accelerated progression to tuberculosis (TB) and to acquired immune deficiency syndrome (AIDS) during coinfection [2,4,5]. Both are responsible for a paradoxical effect observed in coinfected patients after the initiation of antiretroviral therapy (ART), referred to as immune reconstitution inflammatory syndrome (IRIS), a severe local and systemic inflammatory response [6]. Approximately 13 million people are estimated to be coinfected with both pathogens, accounting for 250,000 deaths in 2022, with about 1.3 million new infections by HIV [7] and 1.4 million with Mtb [5]. While antibiotic therapy to treat TB exists, as well as an established ART for controlling chronic HIV infection, the rising resistance to both treatments and drug–drug interactions are posing serious concerns for the effective control of pathogens and instructing an urgent need for new therapies [8,9,10,11,12,13,14,15].

TB is a leading cause of death among HIV-infected people [16]. The interactions between HIV and Mtb that contribute to tuberculosis progression from latency, as a result of the immunosuppressive environment of type I interferons and IL-10, have been the subject of more extensive studies than those that Mtb employs to enhance virus replication and persistence [3,8,17,18,19,20]. A deeper understanding of the pathways underlying these interactions may contribute to the control of both pathogens and the identification of new targets for the development of new efficacious therapeutics, particularly in the context of coinfection.

Our group has investigated the role of lysosomal cathepsins and their inhibitors, cystatins, during Mtb infection [21,22,23] and during HIV coinfection [24,25]. The results revealed that Mtb can block cathepsin proteolytic activity, which contributes to its intracellular survival in macrophages (Mφs) and poor activation of T lymphocytes. Concerning the natural inhibitors, there was a significant increase in gene expression for cystatins C, A, and SN during the early stages of infection, which was evident and common in both Mφ monoinfection and coinfection with Mtb and HIV [24]. Cystatin F (CstF) showed the highest upregulation among the inhibitors, during Mtb monoinfection of Mφs or in cells activated by interferon γ (IFN-γ) [24]. Indeed, we have developed various strategies to overcome the blockade induced by Mtb, including the regulation of gene expression with microRNAs [26] or using RNA silencing for cystatins [23,24,27]. In addition, we have demonstrated that saquinavir, an HIV protease inhibitor used in ART, can impact cathepsin enzymatic activity and overcome the Mtb-induced blockade [28,29]. These results collectively demonstrate the potential of target protease inhibitors to control Mtb infection.

Mtb infects Mφs, where it establishes intracellular niches [30,31,32]. Appropriate immune responses for their intracellular clearance require CD4^+^ T lymphocytes, particularly Th1, as well as other lymphocytes, including CD8^+^ T cells (CTLs) and natural killer cells (NK) [33,34]. Most of the effects on Mtb-infected cells are based on IFN-γ released by those lymphocytes that mediate Mφ activation, leading to a more microbicidal state [35] The cytotoxic effector cells that result from this cytokine-activated infection environment will contribute to the control of the infection via the release of granzyme–perforin-mediated macrophage apoptosis. Infected and newly arrived noninfected Mφs and lymphocytes come into close contact in one structure, the granuloma, which is a hallmark of TB, or in nearby tissues during the establishment of the infection. HIV-1 has a cell tropism for CD4^+^ T cells and establishes intracellular reservoir sanctuaries in Mφs [36]. Although the simultaneous coinfection of Mφs with both pathogens is possible in vitro, it has not been demonstrated in vivo [2,24]. Cytotoxic immune cells, including Th1 cells but mainly NK cells and CTLs, induce the cell death of infected cells mediated by granzyme and perforin, constituting a pivotal response to control viral infections. However, an ineffective viral clearance occurs during HIV infection [37,38,39,40].

One of the most frequent manifestations of TB during HIV coinfection is pleurisy [41]. Both the pleural milieu and the granuloma structure provide the appropriate contact between Mtb-infected cells and noninfected phagocytes with HIV-infected lymphocytes in a particular environment of cytokines and other factors that, all together, help viral replication and spread [42,43]. Surprisingly, CstF levels were found to be significantly increased in the pleural fluids of TB patients compared to other inflammatory conditions [44].

In addition to the previously demonstrated Mtb microbicidal effects, this study aims to assess the potential of cystatin F manipulation to control HIV infection at the coinfection interface. To achieve this, we depleted cystatin F by siRNA in Mφs prior to infection with Mtb and evaluated the trans effects on cultures of peripheral blood-derived lymphocytes infected with HIV. In conclusion, the results of this study indicate potential future directions for controlling both syndemic pathogens through CstF manipulation.

## 2. Results

### 2.1. Experimental Conditions for Transfection and Infection Produce Comparable Low Levels of Cell Death

**Figure 1:**
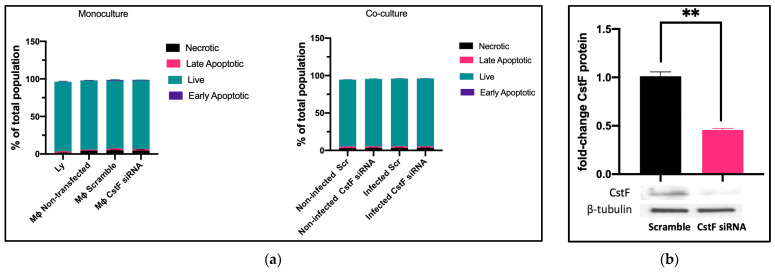
(**a**) Flow cytometry analysis of the percentage of live cells relative to those in programmed cell death (**a**). Apotracker Green (apoptosis), Zombie Red (necrotic cells), and CD3 Alexa Fluor 700 (lymphocytes) dyes were used to stain monocultures for each pathogen or cocultures of Mtb-infected Mφs and HIV-infected lymphocytes. No interference in cell viability was observed on either transfected cells (3 days post-treatment) or infected cells (12 h after infection with pathogens). There were no differences observed in programmed cell death when CstF expression was silenced in Mtb-infected Mφs cocultured with HIV-infected lymphocytes (right panel; coculture conditions). (**b**) The Western blot image demonstrates the silencing of the CstF protein by siRNA at the moment of infection. The respective bar plot was calculated from three independent experiments measuring band intensity using β-tubulin as a calibrator. The error bars represent the standard error of the mean (** *p* ≤ 0.01).

We established the experimental conditions that produce comparable and low levels of cell death between infected and noninfected cells. Mtb-infected Mφs and HIV-infected lymphocytes were analyzed for their effect on cell viability after transfection and 12 h of infection. Infected cells in monocultures were also compared to those in cocultures. Cell death was evaluated by flow cytometry using markers for apoptosis and necrosis, namely Apotracker Green and Zombie Red, respectively. Lymphocytes were distinguished from monocytes using CD3-specific Alexa Fluor^®^ 700 antibodies. Figure 1a shows that there were no differences observed in Mφ monoculture conditions when comparing CstF-silenced phagocytes to cells transfected with scramble or non-transfected. Notably, during coculture, noninfected (scramble) or CstF siRNA-treated cells displayed similar viability to the respective infected conditions (Figure 1a, coculture). Indeed, monocultures of HIV-infected lymphocytes displayed high viability by the end of 12 h p.i. Therefore, it can be concluded that the experimental conditions established here and previously [45] produce cells with similar viability and low impact at the early time points of the coinfection, resulting in low interference for the next coculture assays. CstF was depleted by siRNA prior to the infection of macrophage cultures with Mtb. As shown in Figure 1b, approximately 60% of protein depletion was achieved. The CstF siRNA-treated cells were used in all subsequent experiments, ensuring that the silencing level remained consistent across all conditions assayed.

### 2.2. Decreased CstF Levels from Mtb-Infected Macrophages Are Correlated with Increased Enzymatic Activity of Cathepsin C in Lymphocytes during Coinfection

**Figure 2:**
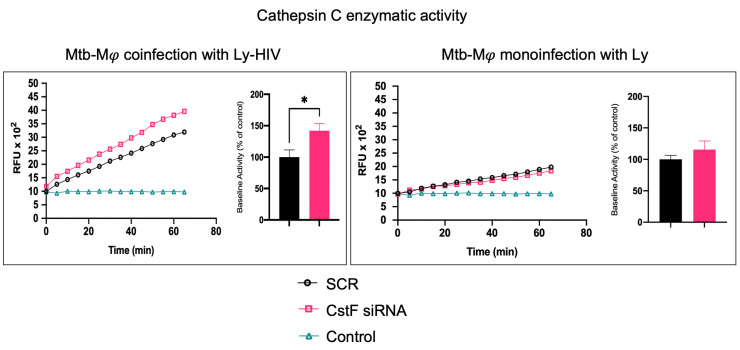
Silencing of CstF expression affects the proteolytic activity of cathepsin C. The enzymatic activity was measured in both scramble control and CstF-silenced cells during cocultures of macrophages with lymphocytes during monoinfection with Mtb or during coinfection with lymphocytes infected with HIV. A cathepsin C-specific fluorogenic substrate was used every 5 min for 60 min. A specific inhibitor was used as a negative control. The bar plots represent the average baseline activity calculated as the largest slope of fluorescence emission over 1 h from three biological replicates. The slope of fluorescence emission in the scramble control was represented as 100%, and each sample’s effect was shown in a percentage relative to the control. The error bars represent the standard error of the mean. The line plots represent fluorescence over time from one representative experiment. (* *p* ≤ 0.01). Mφ, macrophages; Mtb, *Mycobacterium tuberculosis*; RFU, relative fluorescence units.

CstF can be internalized from the extracellular milieu into the endocytic pathway of cytotoxic immune cells, where the inhibition of the pro-granzyme convertase cathepsin C occurs [46,47]. As we previously observed the high expression of CstF during Mφ infection with Mtb and in noninfected phagocytes, activated by IFN-γ [24], here, we depleted CstF using siRNA on infected phagocyte cultures and evaluated its effects on cathepsin C during coculture with lymphocytes. The enzymatic activity was measured by continuously monitoring the formation of fluorescent degradation products using a specific fluorogenic substrate for cathepsin C. A cathepsin-specific inhibitor was used as negative control. Figure 2 shows the significantly higher enzymatic activity of cathepsin C when co-cultivated Mφs were treated with CstF siRNA compared to the respective scramble control during HIV coinfection conditions. No detectable effects on cathepsin C activity in response to CstF silencing were observed for Mφ cocultures infected with Mtb with noninfected lymphocytes (Figure 2). Overall, the results indicate that countering CstF overexpression by depleting CstF in Mtb-infected Mφs impacts the enzymatic activity of cathepsin C in lymphocytes during coinfection with HIV [48,49].

To further elucidate the contribution of noninfected Mφs and Mtb-infected cells as sources of cathepsin C and a potential activation of cytotoxic activity from phagocytes to lymphocytes, we assessed the effects of IFN-γ in substitution of the activation provided by lymphocytes. In this manner, the possibility of lymphocytes serving as a potential source of cathepsin C, as expected in coculture, is eliminated.

**Figure 3:**
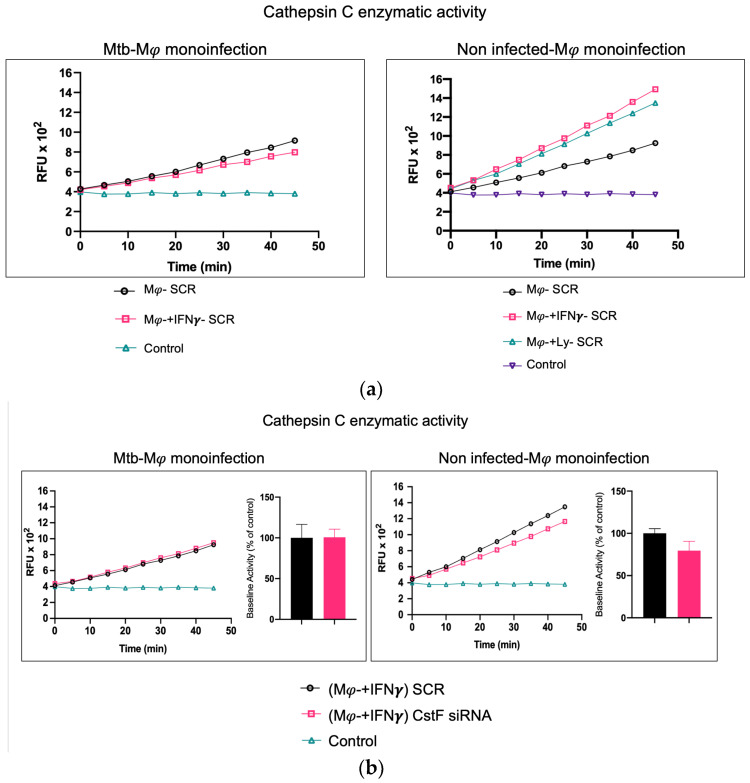
In contrast to noninfected cells, IFN-γ-activated Mφs infected with Mtb do not serve as a source of cathepsin C. (**a**) The enzymatic activity of cathepsin C was compared between resting macrophages and macrophages treated with IFN-γ or in coculture with lymphocytes relative to the control. (**b**) The silencing of CstF expression does not affect the proteolytic activity of cathepsin C in noninfected cells or in cultures infected with Mtb. The enzymatic activity was quantified in both the scramble control and CstF-silenced cells during monocultures of macrophages, which were either activated or not by IFN-γ. A cathepsin C-specific fluorogenic substrate was used at 5 min intervals over a 60 min period. A specific inhibitor was used as a negative control. The bar plots represent the mean baseline activity, calculated as the greatest slope of fluorescence emission over one hour from three biological replicates. The fluorescence emission slope in the scramble control was set at 100%, and the effect of each sample was expressed as a percentage relative to this control. The error bars represent the standard error of the mean. The line plots represent the temporal evolution of fluorescence in a single representative experiment. Mφ, macrophages; Mtb, *Mycobacterium tuberculosis*; RFU, relative fluorescence units.

As shown in Figure 3a, the treatment of noninfected monocultures with IFN-γ resulted in an increase in the enzymatic activity of cathepsin C when compared to the scramble control. However, this activity is not observed in Mtb-infected cells, where IFN-γ treatment results in a further reduction in cathepsin C activity in comparison to the scramble (Figure 3a). This finding corroborates our previous results on gene expression for cathepsin C, which indicated a downregulation of the irrespective gene [21]. Moreover, no differences in cathepsin C activity were observed in monoculture conditions with Mtb infection between resting Mφs, Mφs activated by IFN-γ (Figure 3a), or phagocytes in coculture with noninfected lymphocytes (Figure 2). Furthermore, the depletion of CstF had no impact on the enzymatic activity of cathepsin C in IFN-γ-activated Mφs with Mtb in comparison to the scramble control (Figure 3b).

In contrast, in noninfected cells, a significant effect on cathepsin C activity is observed in IFN-γ-treated cells or in coculture with lymphocytes in comparison to resting Mφs (Figure 3a), with non-significant effects in CstF-depleted cells compared to scramble control (Figure 3b).

It can be concluded that the effect of CstF depletion on cathepsin C activity was only observed in conditions of the coculture of Mtb-infected Mφs with HIV-infected lymphocytes.

### 2.3. CstF Depletion Is Correlated with Increased Cathepsin C–Granzyme B-Driven Cytotoxic Effects

**Figure 4:**
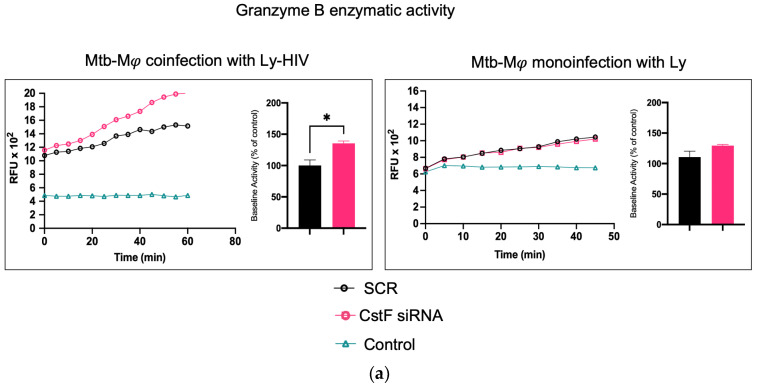

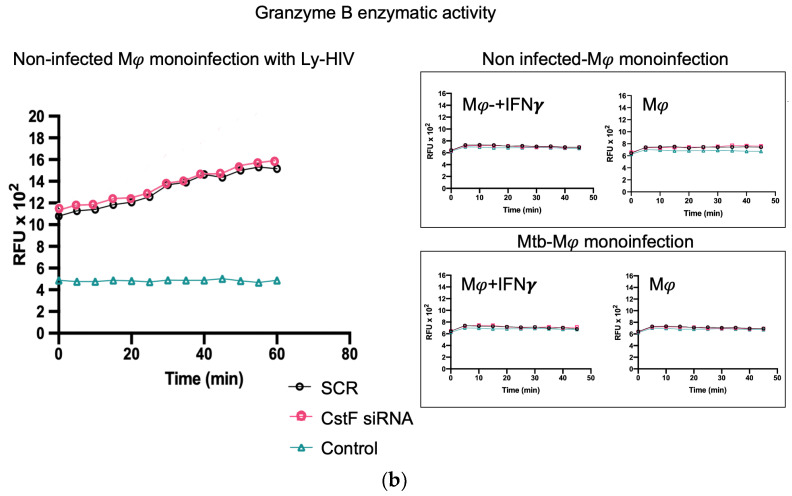
The silencing of CstF expression impacts the cytotoxic activity of granzyme B during Mtb coinfection with HIV. (**a**) The coculture of Mtb-infected macrophages with lymphocytes during coinfection with HIV or during monoinfection with Mtb. (**b**) Effects on monocultures of macrophages, either activated by IFN-γ or not, and on noninfected macrophages in a coculture with lymphocytes infected with HIV. The enzymatic activity of granzyme B was quantified in the cells. This was carried out in both a scramble control and in CstF-silenced cells. A granzyme B-specific fluorogenic substrate was used at 5 min intervals over a 60 min period. A specific inhibitor was used as a negative control. The bar plots represent the mean baseline activity, calculated as the greatest slope of fluorescence emission over one hour from three biological replicates. The fluorescence emission slope of the scramble control was set at 100%, and the effect of each sample was expressed as a percentage relative to this control. The error bars represent the standard error of the mean. The line plots illustrate the fluorescence per time from a single representative experiment. (* *p* ≤ 0.01). Mφ, macrophages; Mtb, *Mycobacterium tuberculosis*; RFU, relative fluorescence units.

It was next investigated whether higher levels of cathepsin C-induced proteolysis led to increased granzyme B activity in cytotoxic lymphocytes. As expected, granzyme B activity was significantly higher in CstF-silenced conditions and coculture conditions during coinfection with HIV compared to the scramble control (Figure 4a). However, no effects were observed during the coculture of Mtb-infected Mφs with noninfected lymphocytes since the depletion of CstF did not impact the granzyme B activity of those cells compared to the scramble control (Figure 4) [50,51,52]. To further confirm that the increased activity of granzyme originates from lymphocytes rather than from Mφs, the effects were evaluated in Mφs activated by IFN-γ in accordance with the results on cathepsin C described above. As shown in Figure 4b, no differences in granzyme activity were observed when comparing IFN-γ-activated Mφs with non-treated controls. Moreover, a significant increase in cathepsin C activity was observed in noninfected phagocytes, which did not result in any discernible impact on granzyme proteolytic activation. Indeed, the depletion of CstF did not affect granzyme activity when it was compared with the scramble conditions. Upon the evaluation of the cocultures of noninfected macrophages with lymphocytes (Figure 4b), no effects on the depletion of CstF were detected. Conversely, a general increase in granzyme activity was observed when lymphocytes were infected with HIV. This is analogous to the increase in granzyme activity observed during Mtb and HIV coinfection (Figure 4a).

We conclude that the depletion of CstF interferes with the granzyme enzymatic activity of lymphocytes exclusively in the context of Mtb–HIV co-infection. The results indicate that the cytotoxic activity is provided through a decreased secretion of CstF from Mφs rather than through granzyme activation in these phagocytes. Indeed, from the results obtained, it may be inferred that the increase in granzyme B is not achieved through a potential effect of CstF depletion on noninfected macrophages, which would potentially lead to an increased secretion of cathepsin C from macrophages to lymphocytes.

### 2.4. CstF Depletion Improves Cathepsin C/Granzyme B-Driven Reduction in Viral Replication During Mtb–HIV Coinfection

**Figure 5:**
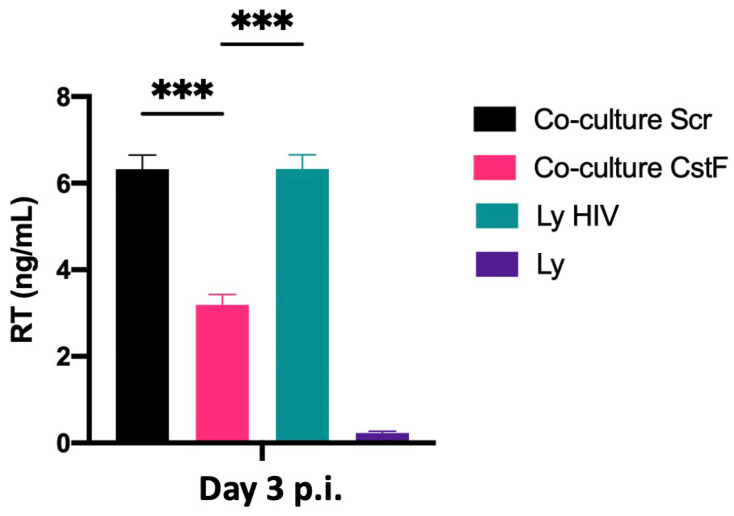
Depletion of CstF in Mtb-infected Mφs enhances cathepsin C/granzyme B-mediated cytotoxic effects on HIV-infected cells during coinfection. Mtb-infected cells, whether CstF-depleted or scrambled, were cocultured with HIV-infected lymphocytes. A significant decrease in viral replication, as determined by RT activity in culture supernatants, was observed in Mtb-infected cells with CstF depletion. HIV-infected lymphocytes were used as a positive control and, as a negative control, we used cells that were not inoculated with HIV. Bar plots represent the average of three biological replicates from one representative experiment performed in duplicate. The error bars represent the standard error of the mean. Scr, scramble (*** *p* ≤ 0.001).

We next aimed to determine whether the increased cytotoxic effects of granzyme B could decrease HIV replication in Mtb–HIV cocultures. As shown in Figure 5, there was a significant decrease in viral particle production at the end of 72 h p.i., as assessed by reverse transcriptase analysis of culture supernatants. Noninfected lymphocytes were used as a negative control.

The results indicate that the manipulation of CstF during coinfection conditions may represent a potential strategy for controlling HIV infection at the interface of the infection with Mtb and HIV.

**Figure 6:**
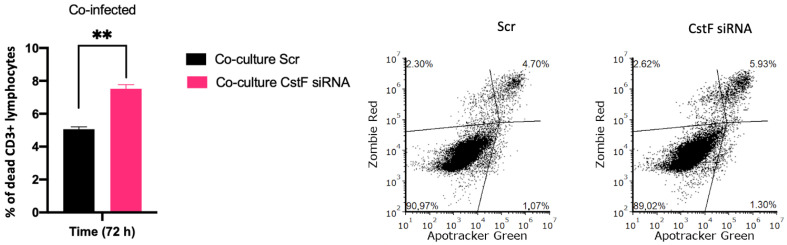
Manipulation of CstF expression during the coinfection of Mtb-infected Mφs with HIV-infected lymphocytes induces granzyme B apoptotic effects. Apotracker Green (apoptosis), Zombie Red (dead cells), and CD3 Alexa Fluor 700 (lymphocytes) dyes were used for staining, and analysis was performed using flow cytometry 72 h p.i. Results represent the mean of three biological replicates (** *p* ≤ 0.01). The error bars represent the standard error of the mean. The dot plots depict representative results from one experimental replicate.

Finally, to provide further evidence on the effects of CstF manipulation on HIV depletion, the impact of granzyme B-driven apoptosis was evaluated. Figure 6 shows that apoptosis was more pronounced in CstF-silenced cocultures of Mtb-infected Mφs and HIV-infected lymphocytes when compared to the scramble control by the end of three days post-infection. This suggests that the depletion of CstF from Mtb-infected Mφs has a significant impact on cathepsin C/granzyme B-driven apoptosis of lymphocytes.

Overall, the results demonstrate that the depletion of CstF in Mtb-infected cells correlates with increased activity of cathepsin C and granzyme B on lymphocytes, resulting in higher cytotoxicity of lymphocytes towards HIV-infected cells and leading to a reduction in viral replication.

## 3. Discussion

Previous work from the group revealed that CstF, a protease inhibitor of lysosomal cathepsins, plays a significant role in Mtb infection, contributing to the intracellular survival of Mtb in human Mφs [27]. The depletion of CstF resulted in the control of the infection, even in clinical strains of Mtb that are resistant to first-line antibiotics used to treat TB [27]. Moreover, a significant increase in the expression of the protease inhibitor during Mtb infection was previously demonstrated, which impacts the proteolytic activity of lysosomal proteases [24]. In contrast, HIV infection did not contribute to an increase in CstF gene expression. These results are consistent with previous studies showing a general decrease in CstF gene expression in CD4^+^ T lymphocytes infected with HIV, as well as in genes related to cytotoxicity [53].

TB remains a significant public health concern, with one of the contributing factors being the synergistic effect of the coinfection with HIV. Although HIV can also infect Mφs, CD4^+^ T lymphocytes are the primary target cells. While our recent work has demonstrated in vitro Mφ infection with both pathogens [24], this has not yet been observed in vivo [2]. The objective of our experiments was to replicate the conditions observed in vivo in the lungs of patients simultaneously infected with Mtb and HIV. Therefore, Mφs were infected with Mtb and cocultured with autologous peripheral blood-derived lymphocytes infected with HIV. To achieve this, lymphocytes were isolated from the blood of healthy donors, including CD4^+^ and CD8^+^ naive T lymphocytes and conventional and unconventional NK cells. Since the Portuguese population has been vaccinated for BCG until the last 5 years, it is expected that PBMCs from healthy donors also contain effector and memory T lymphocytes that recognize autologous Mφs infected with Mtb.

CstF was shown to be secreted from immune cell producers into the extracellular milieu and internalized by bystander cells [46,54,55,56]. A key target of the protease inhibitor is cathepsin C, a major progranzyme convertase [57]. Likewise, the internalization of CstF was observed to have an inhibitory effect on cytotoxic cells, both in NK [46] and in CD8^+^ cytotoxic T lymphocytes (CTL) [47], leading to anergy split, a condition where these cells lose the ability to secrete granzyme and perforin [57]. Additionally, human NK cells displayed a 30-fold increase in CstF compared to CTL [58]. However, it was not demonstrated whether this difference resulted from the accelerated synthesis and/or increased internalization of secreted CstF by closely interacting immune cells [57].

In this study, we depleted CstF from Mφs prior to Mtb infection by using siRNA, based on our previous results, indicating a high gene expression of CstF from Mφs activated with IFN-γ and/or in Mtb-infected phagocytes [21]. We designed an in vitro experiment to assess whether the manipulation of CstF could contribute to the control of HIV replication during coinfection with Mtb.

As in previous work, here we provided evidence of successful CstF silencing at the protein synthesis level [27]. The current results indicate that the depletion of CstF from Mtb-infected Mφs enhances cathepsin C activity in cocultured lymphocytes, thereby augmenting their granzyme cytototoxic effects. However, no effects of CstF depletion were observed in an autocrine manner in activated Mφs with regard to cathepsin C or granzyme B. Such effects are observed in monocultures of IFN-γ-stimulated noninfected Mφs or during Mtb infection. Indeed, while Mφs potentially can be activated by IFN-γ to a cytotoxic phenotype under certain conditions by activated effector Th1 lymphocytes [48,49,50,51,52], here, no higher granzyme activities were observed following CstF depletion or after IFN-γ activation in Mφs. The results suggest that the observed increase in granzyme activity is driven by cytotoxic lymphocytes in coculture, rather than by activated Mφs. Since an increase in cathepsin C was observed in noninfected Mφs following IFN-γ stimulation, but not in Mtb-infected Mφs, it can be hypothesized that noninfected Mφs in culture conditions may contribute via cathepsin C secretion to lymphocytes, thereby driving granzyme activation and subsequent internalization in bystander cells. Nevertheless, no effects on CstF depletion were observed in either monocultures of Mφs or cocultures of noninfected Mφs with lymphocytes infected with HIV.

We conclude that the observed increase in cathepsin C enzymatic activity during coculture assays in Mtb–HIV coinfection is a result of the activation of this enzyme from cytotoxic lymphocytes following the depletion of CstF from Mtb-infected Mφs. This activation does not occur in noninfected phagocytes. Ultimately, the depletion of CstF in noninfected Mφs may result in an increased activity of cathepsin C in phagocytes, and their secretion could potentially lead to granzyme activation in lymphocytes. However, no effect of CstF depletion was observed in monocultures or in cocultures with lymphocytes from noninfected cells.

It can be inferred that a decrease in cathepsin C and an increase in CstF secretion, induced by Mtb, will result in the evasion of cytolytic activity by lymphocytes directed to Mtb-infected cells. This scenario will not be observed in noninfected phagocytes.

**Figure 7:**
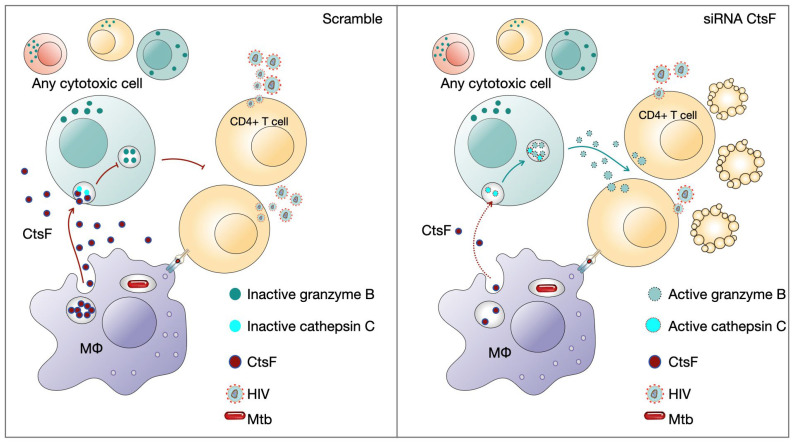
CstF depletion in Mtb-infected Mφs improves cathepsin C/granzyme B-driven cytotoxic effects on HIV-infected cells. Cytotoxic lymphocytes are potential targets to be affected by CstF secreted from Mtb-infected Mφs.

HIV infection can evade the early immune response, resulting in ineffective viral clearance. Cytotoxic lymphocytes including NK cells and HIV-specific adaptive cells such as CTLs or even Th1 are crucial for the outcome of infection and arise shortly after infection [1,40]. Additionally, high levels of NK recruitment have been observed in tuberculous pleural effusions and in early innate granulomas [59]. Pleurisy is a common manifestation of TB, often observed during the primo infection [41]. It is also frequently observed in HIV-coinfected patients, where high levels of virus particles are present at the sites of Mtb infection [42,43]. Coincidently, higher levels of CstF were found in pleural effusion of TB patients than in other inflammatory conditions [44]. Here, our results indicate that in vitro, when this environment of cells and cytokines is reproduced, a clear impact of CstF depletion from Mtb-infected macrophages increases the cytotoxic activity of lymphocytes, with consequences on HIV replication and viral loads (Figure 7).

The results also suggest the existence of an evasion mechanism that enables early HIV replication during coinfection with Mtb at the interface environment through the CstF/cathepsin C/granzyme B axis. Ultimately, this knowledge can be crucial for developing new therapeutic approaches to control both pathogens based on the manipulation of CstF.

## 4. Materials and Methods

### 4.1. Cell Isolation and Culture Conditions

Primary human monocyte-derived Mφs were isolated and then differentiated from anonymous buffy coats of healthy human donors, which were provided by the National Blood Institute (Instituto Português do Sangue e da Transplantação, I.P., Lisbon, Portugal) following a previously described protocol [24]. Autologous lymphocytes were obtained from the peripheral blood mononuclear cell (PBMC) fractions by lysing red blood cells. The lymphocytes were then stimulated with 3 μg/mL of Phytohemagglutinin-L (PHA-L) (ThermoFisher, Waltham, MA, USA) for three days prior to infection with HIV. They were further cultured in a 75 cm^2^ flask at 2 × 10^6^ cells per mL in a Roswell Park Memorial Institute (RPMI) medium (RPMI-1640) (Hyclone, GE Healthcare, Hertfordshire, UK) supplemented with 15% (*v*/*v*) Fetal Bovine Serum (FBS) (Hyclone, GE Healthcare), 2 mM L-glutamine (Gibco), and 20 UI/mL of human recombinant interleukin-2 (BioLegend, San Diego, CA, USA). IL-2 or M-CSF were not further added during coculture procedures. IFN-γ was used at a concentration of 100 IU/mL for the activation of Mφs when required.

### 4.2. Bacterial Cultures and HIV Isolates

*M. tuberculosis* H37Rv (ATCC 27294) (American Type Culture Collection, Manassas, VA, USA) (Mtb) was grown in Middlebrook’s 7H9 medium supplemented with 10% Oleic acid–Albumin–Dextrose–Catalase enrichment (OADC) (Difco, Omagh, UK), 0.02% glycerol, and 0.05% tyloxapol at 37 °C. The primary HIV-1_UCFL1032_ isolate was obtained by coculturing PBMCs isolated from the infected patient with PBMCs from uninfected individuals, as described [45]. After isolation, viral stocks were established in PBMCs from low-passaged supernatants of original cultures and stored at −80 °C until further use. All experimental procedures using Mtb and HIV were performed in the biosafety level 3 laboratory at the Faculty of Pharmacy of the University of Lisbon, maintaining the national and European containment level 3 laboratory management and biosecurity standards based on applicable EU directives.

### 4.3. Macrophage Infection

Before infection, Mtb was cultivated at 37 °C, 5% CO_2,_ until the exponential growth phase was reached. On the day of infection, the bacterial suspensions were treated for clump removal and a single cell was obtained, as described before [23,24,27], centrifuged, washed in phosphate-buffered saline (PBS), and resuspended in RPMI culture medium without antibiotics. Clumps of bacteria in the suspension were disrupted by ultrasonic bath treatment for 5 min and removed by centrifugation at a low speed of 500× *g* for 1 min. The obtained single-cell suspension was verified by fluorescence microscopy and quantified by measuring optical density at 600 nm. The infection was performed with a multiplicity of infection (MOI) of 1 bacterium per Mφ for 3 h at 37 °C, 5% CO_2_. Following this incubation period, cells were washed with PBS to remove free bacteria and added to a fresh complete medium.

### 4.4. Transfection

Mφs were transfected 72 h before infection to achieve maximum RNA silencing. Transfection with anti-CstF siRNA or with scramble control siRNA was performed with ScreenFect A (ScreenFect GmbH, Eggenstein-Leopoldshafen, Germany) transfection reagent according to the manufacturer’s protocol, as previously described [27]. Mφs were incubated for 72 h with the transfection reagent and SMARTpool ON-TARGETplus Human CST7 siRNA (Dharmacon, Lafayette, CO, USA; target sequences: AGUGAAAGGCCUGAAAUAU, GAAAUUGGCAGAACUACCU, GGAUGACUGUGACUUCCAA, and CAAGGGCCCUAGUUCAGAU) or the respective siRNA non-targeting (scramble) control (Dharmacon, USA; target sequences: UGGUUUACAUGUCGACUAA, UGGUUUACAUGUUGUGUGA, UGGUUUACAUGUUUUCUGA, and UGGUUUACAUGUUUUCCUA) in the medium without antibiotic. The same transfected or scrambled cells were split and used for the whole required further experiments.

### 4.5. Enzymatic Activities of Cathepsin C and Granzyme B

After 48 h of infection, Mφ cultures in 96-well plates were lysed with chilled lysis buffer, 25 mM 2-(N-morpholino)ethanesufonic acid (MES) (MP Biomedicals, Singapore), 100 mM NaCl, and 5 mM cysteine, pH 6 for cathepsin C, and 50 mM Tris-HCl and 100 mM NaCl, pH 7.4 for granzyme B. Cells were centrifuged at 16,000× *g* for 20 min at 4 °C to recover the supernatant and further added to reaction buffer for 15 min at room temperature for cathepsin C or for 30 min at 37 °C for granzyme B. The specific fluorogenic substrates, 70 μM H-Gly-Phe-7-amino- 4-methylcoumarin (AMC) (Bachem, Bubendorf, Switzerland) for cathepsin C and 50 μM acetyl-Ile-Glu-Pro-Asp-AMC for granzyme B (Bachem), were then added, and the formation of fluorescent degradation products was measured continuously with excitation at 370 nm and emission at 460 nm in a Tecan M200. The activity of the control sample was set to 100% and activities for the other samples were adjusted accordingly. Noninfected Mφs were subjected to the same treatment for enzymatic activity assessment.

### 4.6. Western Blotting

Total proteins were harvested using a RIPA buffer (Merck, KGaA, Darmstadt, Germany). The protein samples were diluted 1:1 in Laemmli buffer (Merck, KGaA, Darmstadt, Germany) and heated at 95 °C for 5 min before running the gel. The protocol followed the same as described in [27]. Proteins were subjected to sodium dodecyl sulfate–polyacrylamide gel electrophoresis (SDS–PAGE) using 4–20% Mini-PROTEAN TGX Precast Protein Gels (Bio-Rad Laboratories, Hercules, CA, USA) and transferred to the nitrocellulose membrane through the Trans-Blot Turbo Transfer System (Bio-Rad Laboratories, Hercules, CA, USA). Furthermore, the membrane was processed and stained using the iBind Western system (Thermo Fisher Scientific, Waltham, MA, USA) and primary antibodies specific for CstF (1:2000 dilution of #SAB2700222, Sigma-Aldrich), ß-tubulin (1:4000 dilution of #ab6046, Abcam, Cambridge, UK), and horseradish peroxidase (HRP)-conjugated secondary antibody (1:4000 dilution of #1706515, Bio-Rad Laboratories, Hercules, CA, USA). The visualization of bands was performed through chemiluminescence using an NZY Supreme ECL HRP Substrate (NZYTech, Lisbon, Portugal) in a ChemiDoc XRS+ System (Bio-Rad Laboratories, Hercules, CA, USA). The quantification of band intensity was performed using ImageLab software version 6.1, on USB drive #12012931 (Bio-Rad Laboratories, Hercules, CA, USA).

### 4.7. Lymphocyte Infection with HIV and Coculture with Macrophages Infected with Mtb

Autologous lymphocytes were obtained from the PBMC fractions, stimulated, and further cultured according to the protocol described above. On the day of infection, lymphocytes were infected with 1000 TCID50/mL of HIV-1_UCFL1032_ or left uninfected as controls. Briefly, viruses were added and incubated for 3 h in the presence of 3 μg/mL of polybrene (Sigma-Aldrich, St. Louis, MO, USA). Cells were then washed with PBS to remove any unadsorbed virus particles and cultured in an appropriate medium (500 μL/well). Mφs were allowed to internalize Mtb for 3 h. After this chase period, they were washed with PBS to remove extracellular bacteria and cocultivated with the HIV-infected lymphocytes at a ratio of 1:2. Culture supernatants were collected at days 3 and 9 to recover virus particles and quantified by reverse transcriptase activity.

### 4.8. HIV Quantification

Supernatants collected from cocultures with lymphocytes infected with HIV, as described above, were used for viral replication quantification. This was assessed by using a colorimetric enzyme immunoassay (Roche, Merck KGaA, Darmstadt, Germany) for the quantitative determination of retroviral reverse transcriptase activity by the incorporation of digoxigenin- and biotin-labeled dUTP into DNA. Absorbance was measured by Tecan M200 spectrofluorometer at 405 and 490 nm.

### 4.9. Cell Death and Viability Assays Using Flow Cytometry

For the assessment of apoptotic or necrotic cells, the Apotracker Green and Zombie Red (Biolegend, San Diego, CA, USA) dyes were used, respectively. Mφs were allowed to internalize Mtb and lymphocyte HIV particles for 3 h. After the internalization step, extracellular bacteria were removed by washing Mφ cultures with PBS, and extracellular viruses were eliminated as described previously. Monocultures or cocultures were further incubated for additional timing with the recommended cell death kit experiments reagents and evaluated at the end of 12 h or after 3 days of treatment. The corresponding noninfected cells treated or not with transfection reagents and siRNAs were evaluated in parallel using the same kit. After those timings, cultured cells were detached using 5 mM EDTA. Human peripheral blood lymphocytes were stained with Alexa Fluor^®^ 700 anti-human CD3 antibody (Biolegend, San Diego, CA, USA). Cells were fixed in 4% paraformaldehyde for one hour and then analyzed in a Cytek^®^ Aurora flow cytometer (Cytek^®^ Biosciences, Fremont, CA, USA). Data analysis was performed in FCS Express 7 (De Novo Software, Pasadena, CA, USA).

### 4.10. Statistical Analysis

Statistical analysis was performed in GraphPad Prism 9. Multiple group comparisons were conducted using one-way ANOVA followed by a Holm–Sidak post hoc test. Two group comparisons were made using Student’s *t*-test. Differences were considered statistically significant when the calculated adjusted *p*-value was equal to or below the alpha level of 0.05 (*p* ≤ 0.05).

## Data Availability

Data is contained within the article.
